# The Interplay between Magnesium and Testosterone in Modulating Physical Function in Men

**DOI:** 10.1155/2014/525249

**Published:** 2014-03-03

**Authors:** Marcello Maggio, Francesca De Vita, Fulvio Lauretani, Antonio Nouvenne, Tiziana Meschi, Andrea Ticinesi, Ligia J. Dominguez, Mario Barbagallo, Elisabetta Dall'Aglio, Gian Paolo Ceda

**Affiliations:** ^1^Section of Geriatrics, Department of Clinical and Experimental Medicine, University of Parma, Via Gramsci 14, 43100 Parma, Italy; ^2^Geriatric Rehabilitation Department, University-Hospital of Parma, Via Gramsci 14, 43100 Parma, Italy; ^3^Department of Internal Medicine and Medical Specialties (DIMIS), University of Palermo, Piazza delle Cliniche 2, 90127 Palermo, Italy

## Abstract

The role of nutritional status as key factor of successful aging is very well recognized. Among the different mechanisms by which nutrients may exert their beneficial effects is the modulation of the hormonal anabolic milieu, which is significantly reduced with aging. Undernutrition and anabolic hormonal deficiency frequently coexist in older individuals determining an increased risk of mobility impairment and other adverse outcomes. Mineral assessment has received attention as an important determinant of physical performance. In particular, there is evidence that magnesium exerts a positive influence on anabolic hormonal status, including Testosterone, in men. In this review we summarize data from observational and intervention studies about the role of magnesium in Testosterone bioactivity and the potential underlying mechanisms of this relationship in male subjects. If larger studies will confirm these pivotal data, the combination of hormonal and mineral replacements might be adopted to prevent or delay the onset of disability in the elderly.

## 1. Biological Role of Magnesium

Magnesium is an essential ion involved in multiple fundamental physiologic functions in humans [1]. As part of the activated MgATP complex, magnesium is involved in the pathways generating adenosine triphosphate (ATP) and energy in mitochondria, electron transport chain and complex subunits, and oxygen detoxification. Magnesium is also a cofactor in over 300 enzymatic reactions and biological processes, including protein and nucleic acid synthesis, and neuromuscular excitability [1].

Circulating magnesium exists in three forms. The metabolically active free ionized fraction (magnesium ion) is the most represented, accounting up to 60–70% of the total serum magnesium. Other main serum forms include the protein-bound (25% and 8% bound to albumin and globulin, resp.) and the chelated magnesium fraction (12%) [2].

The most represented reservoir of magnesium in human body is the mineral phase of the bone that accounts for about 64% of total magnesium. The remaining amount is located in the intracellular (34%) and extracellular spaces (1%). The intracellular magnesium concentration is fundamental to ensure the most important cellular and metabolic activities [3]. Indeed, the rapid requirements of this cation are usually met by intracellular stores that more quickly exchange magnesium with intracellular fluids.

Because of the lack of clinical tests available for assessing total-body magnesium content, the serum magnesium concentration remains the most clinically reliable test.

Adequate serum magnesium levels (normal ranges: 0.75–0.95 mmol/L or 1.7–2.5 mg/dL) seem to be critical in ensuring the normal cellular homeostasis [1, 2]. Magnesium status is influenced by dietary intake, absorption in the gastrointestinal tract, renal excretion, and tissue uptake and utilization (e.g., cardiac and skeletal muscle tissue) [4]. To guarantee an optimal magnesium homeostasis the recommended intake from dietary sources is estimated in 420 and 320 mg/day for healthy men and women, respectively [5]. Food-rich magnesium sources are cereals, green leafy vegetables, seeds, nuts, cocoa, and seafood [6]. However, the definition of magnesium deficiency is notoriously complex. Magnesium serum concentrations below the laboratory reference range of <1.8 mg/dL are currently used to define some degree of magnesium depletion. However, this cut-off value could be not necessarily related to a pathophysiologic state of deficiency, because low intracellular magnesium has been documented even in patients with serum magnesium concentrations >1.8 mg/dL [7].

## 2. Magnesium and Muscle Function in Young Trained Individuals

Magnesium has been the most investigated mineral involved in muscle function. The beneficial effects of magnesium on skeletal muscle and physical performance are linked to its known actions on energetic metabolism (phosphorylation processes and reactions requiring ATP, energy utilization and transfer, and transmembrane transport) which have enormous implications in muscle contraction [1]. In fact, dietary magnesium deprivation is associated with increased oxygen requirements to complete submaximal exercise and reduced endurance performance [8].

Magnesium administration elicited the reductions in heart rate, ventilation, oxygen uptake, and carbon dioxide production during submaximal work [9, 10]. In male athletes, 25 days of magnesium (390 mg/d), with a 3 wk washout, increased peak oxygen uptake and total work output during work capacity tests [11]. Similarly, in physically active collegians, magnesium supplementation significantly improved endurance performance and oxygen utilization [12]. In a depletion-repletion experiment in 10 postmenopausal women (aged 45–71), dietary magnesium (320 versus 180 mg/d) improved magnesium balance, erythrocyte and skeletal muscle magnesium concentrations, heart rate, and oxygen consumption during submaximal exercise [13].

It is not surprising that most of the current observational and intervention studies have been conducted on athlete subjects. In young men participating at 7-week strength training program, supplemental magnesium was capable of significantly improving muscle strength and power [14]. The gain in muscle strength occurred at dietary magnesium intake higher than 250 mg/d and was even more evident at 500 mg/d (exceeding the recommended dietary allowance, RDA) [14]. However, magnesium supplementation per se does not affect work performance in magnesium-replete trained individuals [15]. Dietary surveys reveal a magnesium intake equaling or exceeding the RDA for male athletes [16]. In female athletes it tends to be 60% to 65% of the current recommendation. Regardless of sex, athletes competing in sports requiring weight classifications or esthetic components tend to consume up to 30–55% of the magnesium RDA [17]. Serum magnesium levels may also be reduced during intense and/or long-term exercise [18] leading to latent fatigue and decreased endurance [19], similarly to what has been observed during the condition of zinc deficiency [20].

These lines of evidence led to consider magnesium as potentially limiting element for human physical performance, creating the rationale for the routine use of magnesium supplementation during intense endurance exercise.

## 3. Age-Related Changes in Magnesium Levels and Physical Performance

Suboptimal magnesium status is a frequent condition in older persons. The most common cause of magnesium deficit is the low dietary magnesium intake [21]. This is a well-represented phenomenon in older population, occurring in up to 10–15% of community-dwelling older subjects [22]. The typical western diet, highly rich in processed foods and deficient in green vegetables and whole grain, may also contribute to an inadequate magnesium intake.

The magnesium requirement for older population does not differ from young and adult subjects. However, data from the National Health and Nutrition Examination Survey (NHANES) III show an average daily magnesium intake dramatically below the recommended RDA, approximately of 225 mg/day in men and 166 mg/day in women [5].

A suboptimal magnesium status may also result from altered magnesium absorption and/or increased urinary loss [23]. Polypharmacotherapy (loop diuretics, digitals, and proton pump inhibitors) as well as a wide range of clinical conditions (HIV, type 2 diabetes, alcoholism, and cardiovascular diseases) plays additional important roles in lowering magnesium levels [24–26].

However, the magnesium deficiency is more difficult to be detected in the elderly population. In fact the pauperization of intracellular stores is not usually accompanied by a parallel decline in magnesium serum concentrations that tend to remain more stable, within the normal range [27]. The deficiency of magnesium at cellular level and in the body stores is crucial for maintaining the skeletal muscle efficiency. It is very well known that sarcopenia (recently defined by consensus documents as the presence of both low muscle mass and low muscle function (strength or performance)) [28–30] frequently leads to a condition of decreased physiological reserve, increased vulnerability to stressors, and adverse outcomes, known as “frailty” [31]. The frail status is a strong predictor of mortality, independent of traditional indicators of disease [32]. Despite the role of magnesium in muscle integrity and function, there are few data in this regard in the elderly. In a representative cohort of 1138 older men, Dominguez and colleagues [33] using data from the InCHIANTI Study showed a significant, independent, and strong positive relationship between circulating magnesium levels and measures of muscle performance (hand grip strength, lower-leg muscle power, knee extension torque, and ankle extension strength). These authors suggested the need of identifying serum magnesium cut-off values to attain the best possible physical function. These data suggest the potential contribution of low magnesium status, frequently observed in the elderly, to the reduced physical performance.

## 4. The Concept of Nutritional Modulation of Anabolic Hormonal Status in Older Men

In healthy adult subjects, changes in food consumption and utilization may induce homeostatic adaptations that redistribute nutrients without affecting muscle function and physical performance. During the aging process the body energy delivery system could be impaired because of the decline in physiological reserves and the disruption of metabolic pathways, and sarcopenia may arise. Physiological, psychological, and hormonal systems interact to determine the energy need. Macronutrients are essential to provide the body structure to perform work. Minerals are fundamental to enable the use of macronutrients for all physiological processes. In fact, an insufficient qualitative and quantitative nutrient intake is one of the multiple causes of loss of muscle mass, decreased physical performance, and adverse outcomes [34]. Anabolic hormones, whose levels decrease with age, play an important role in maintaining the optimal body energy delivery. In older persons, the occurrence of a single mild hormonal derangement is rarely observed. More frequently there is a simultaneous anabolic hormonal deficiency [Testosterone (T), Dehydroepiandrosterone (DHEA), estradiol (E2), growth hormone-Insulin-like Growth Factor-1 (GH-IGF-1), and vitamin D] which is part of “multiple hormonal dysregulation” [35]. These hormones interplay in ensuring overall anabolic state and induce the satellite cell activation together with exercise and muscle hypertrophy [35]. The simultaneous presence of low levels of Testosterone, together with DHEAS and IGF-1, has a strong effect on all-cause mortality in older men [36]. Experimental data confirm that hormonal therapies, singularly or in combination, may improve body composition and physical performance [37–41]. The nutrients (especially the minerals magnesium, selenium, and zinc) and the anabolic hormones, especially T and IGF-1, seem to interact. The combination of nutritional and hormonal strategies in frail undernourished older people determines a more effective reduction in the number of hospitalizations, the time to hospital admission, and the days of hospital stay [42, 43]. The specific actions of both micronutrients and hormones at skeletal muscle level have led to the hypothesis of an interaction of these factors in ensuring optimal physical performance [44]. This novel concept could have important clinical implications in the elderly, who are more prone to a disruption of the anabolic/catabolic equilibrium and undernutrition. The use of specific mineral supplements may represent a sort of preventive measure of mobility impairment.

## 5. Changes in Testosterone Secretion with Age and Implications in Skeletal Muscle Function 

Testosterone is the most important male sex steroid, synthesized by the Leydig cells of the testes (95%) and derived by peripheral adrenal androgens conversion for the remaining 5% [45].

In men, up to 44–65% of the circulating plasma T is bound to sex hormone binding globulin (SHBG) and 33–54% to albumin, and approximately 2-3% is available as a free form. The free fraction of circulating T plus albumin-bound T represents the amount of biologically active T (Bio-T) that more accurately reflects the clinical androgen state of the subject [46]. Total T levels progressively decrease from the age of 35, by 1% per year. The decline is more pronounced for Bio-T, 2% per year [45], especially in untreated depressed men [47, 48]. The causes of the age-related fall in total and Bio-T levels include a decrease in testicular function and a disruption of hypothalamic-pituitary axis. This peculiar phenomenon of the ageing process involves the GnRH secretion and activity (reduced amplitude of the peaks, attenuation of the circadian rhythm, and reduced sensitivity to negative feedback), the pituitary gland (reduced gonadotropin response to GnRH), and the Leydig cells (reduced response to human chorionic gonadotropin, HCG) [49].

Other mechanisms such as the increased SHBG levels and T aromatization (increased activity aromatase in adipose tissue) as well as the reduced bioconversion of T into dihydrotestosterone could also concur to impair T biological activity with age [50, 51]. Some authors hypothesize that the age-related decline in androgenic activity could be related to the reduced DHEA secretion, which is an important precursor of T [52]. Finally, qualitative changes in signaling transduction (reduced T receptors expression and/or impaired T binding capacity in the liver, brain, and prostate) should be also accounted for [53, 54].

The anabolic, anticatabolic, and neurotrophic effects of T administration on muscle are well known and extensively studied.

Observational studies on castrated animals [55] and adult men [56, 57] show that low T levels are associated with a reduction in lean body mass and muscle strength and other negative changes in body composition.

In a very recent cross-sectional analysis of 250 patients, 70 years or older, Ucak et al. [58] have found a negative impact of “compensated” or “subclinical” hypogonadism (defined as mild biochemical alterations accompanied by signs and symptoms of T deficiency) [59] on physical function, mood, cognitive, and nutritional status.

Intervention studies on elderly subjects have documented beneficial effects of T on counteracting the age-related changes of body composition and physical function [60–62]. T exerts direct influence on lean body mass and strength [63, 64], whereas equivocal evidence is available on the effects of T on physical performance and quality of life [35].

The anabolic effects of T are even more evident in older subjects with mobility limitation.

In 209 community-dwelling men with low T levels (100 to 350 ng/dL [3.5 to 12.1 nmol/L]) from Testosterone in Older Men (TOM) with Mobility Limitations Trial, the daily T gel therapy for 6 months improved both leg-press and chest-press strength and stair-climbing power [65]. Testosterone may also influence muscle metabolism by improving haemoglobin levels in older men with mild anaemia [66, 67]. In women, skeletal muscle tissue seems to be sensitive to the anabolic action of androgens [68]. However, the impact of T administration on full physical function has not been fully studied. The precise molecular mechanisms underlying these observed physical changes in men are likely to include specific T effects on adipocytes and skeletal muscle cell receptors. The binding of T to its receptors could lead to the stimulation of lipolysis and protein synthesis [41, 69]. Finally, several lines of evidence support the hypothesis of permissive effects of T on the differentiation of the precursor stromal cells into muscular line [70].

## 6. The Interplay between Magnesium and Testosterone in Physical Function 

**Figure 1:**
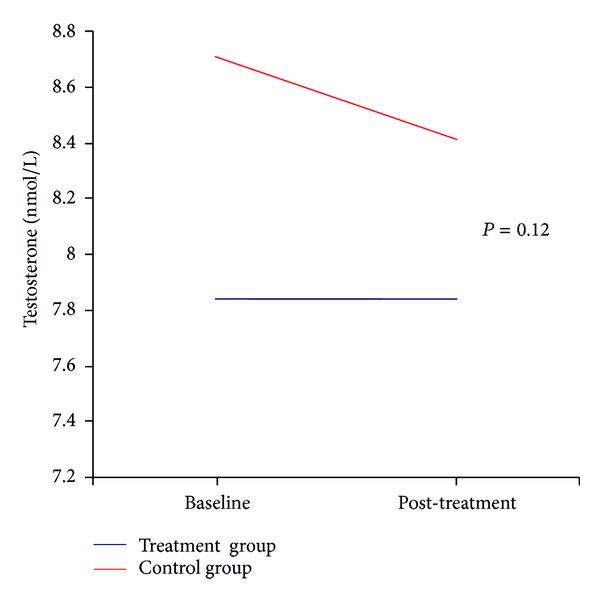
Changes in Testosterone levels at baseline and after treatment. Changes in the average Testosterone levels (nmol/L) before (baseline) and after (post-treatment) magnesium sulfate administration (blue line) and placebo (red line). The difference in total Testosterone levels between the 2 groups touched the statistical significance (*P* = 0.12).

The hypothesis of a link between magnesium and T has been tested in pivotal experiences using magnesium supplementation in adult subjects. Brilla and Conte investigated the combined role of magnesium supplementation and exercise on T levels [71]. A simple zinc-magnesium nutritional formulation (30 mg zinc monomethionine aspartate, 450 mg magnesium aspartate, and 10.5 mg of vitamin B-6) was able to improve T levels of athletes engaging in intense physical activity compared to placebo (132.1 to 176.3 pg/mL versus 141 to 126.6 pg/mL). The highest levels of T were found in those athletes both exercising and receiving magnesium supplementation. Moreover, significant differences in muscle strength via torque measurements and functional power were noted between the 2 groups (189.9 to 211 Nm at 180°/s and 316.5 to 373.7 Nm at 300°/s versus 204.2 to 209.1 Nm at 180°/s and 369.5 to 404.3 Nm at 300°/s). These data have been confirmed in a recent study performed on young subjects, where 4-week magnesium supplementation (magnesium sulfate 10 mg/kg/d) and exercise increase free and total T concentrations at exhaustion before and after supplementation compared to resting levels [72, 73]. There are limited data about the relationship between magnesium and T in study population, especially of older subjects. Maggio and colleagues [74] in 399 older men ≥ 65 years (74.18 ± 6.43 mean age ± SD) from the InCHIANTI Study documented for the first time the strong and positive association between magnesium levels and total T and total IGF-1 levels. Interestingly, the relationship between magnesium and T was independent of body mass index, IL-6, DHEAS, SHBG, insulin, total IGF-1, grip strength, Parkinson's disease, and chronic heart failure. Because of the cross-sectional nature of the study the authors could not establish a cause-effect relationship between magnesium and T levels. This finding led the authors to perform a pilot single-center, randomized, placebo-controlled, single-blind intervention study. 46 elderly hospitalized male subjects (21 in the treatment group), aged 65 years or older, with magnesium serum levels < 2.5 mg/dL, were randomly assigned to magnesium sulfate treatment (1 g/mL of ion Mg++ diluted in 250 cc of normal saline solution) or placebo (250 cc of saline solution) [75]. The active product or placebo was in a single intravenous dose administered in about 30 minutes. Testosterone, IGF-1, SHBG, and C-reactive protein (CRP) concentrations were evaluated before and after treatment. All measurements were performed at the Laboratory of the University-Hospital of Parma. Baseline characteristics between intervention and control groups were analyzed by *t*-test. Paired *t*-test was used to examine and to compare the response trends between the two groups at baseline and after treatment. As expected, magnesium sulfate administration induced a significant increase in serum magnesium levels (delta 1.28 ± 0.61) compared to placebo (delta −0.03 ± 0.14) (*P* < 0.001). Interestingly, total T levels remained substantially unchanged (delta 0.01 ± 0.80) in the intervention group while they were significantly decreased in the placebo group (delta −0.03 ± 0.14). The difference in total T levels between the 2 groups touched the statistical significance (*P* = 0.12) (Figure 1). No differences were appreciated in bioavailable T (Bio-T), IGF-1, and SHBG concentrations. In this preliminary analysis no differences in CRP levels were observed between the two groups at baseline. The adjustment for CRP levels at baseline did not affect the relationship between magnesium and total T [75].

These preliminary data in humans are supported by experimental studies in animal models.

Interestingly, magnesium supplementation has been shown to have an apparent beneficial effect on male gonadal system, as observed in a very recent study performed on sexually mature male rats [76]. Chandra et al. evaluated the morphological, cytological, and functional changes in testis after magnesium administration. Interestingly, the authors showed significant enhancing in steroidogenic enzymes, namely, delta(5)3beta-hydroxysteroid dehydrogenase and 17beta-hydroxysteroid dehydrogenase, activities at moderate and high dose of magnesium that resulted in increased serum T levels [76]. This phenomenon was followed by a progressive development in cytoarchitecture of genital organs without significant changes in quantitative spermatogenesis. The results were remarkably more evident in the groups treated for a longer period and at high doses of magnesium. In mice, dietary magnesium depletion seems to target apical cells within caput epididymis [77]. Moreover, in older male dromedary camel, the age-related decline in plasma T concentrations has been associated with a disruption of the mechanisms controlling normal cation distribution (including magnesium) in the testis, epididymis, and accessory glands [78].

## 7. The Role of Inflammation in the Interplay between Magnesium and Testosterone Levels

We can account for different factors influencing both T and magnesium concentration in adult and elderly men. Particular attention deserves the role of inflammation, which is a negative modulator of both these factors.

Inflammatory cytokines act as inhibitory factors at pituitary (on the secretion of LH) and testicular level (reduction of T secretion and sensitivity of T to LH) [79], contributing to the development of hypogonadism.

Higher levels of inflammatory markers and low T concentration are strong predictors of frailty, disability, and cardiovascular events [80, 81] that negatively influence the muscle mass [82].

In older men, there is a steeper decline of T levels during a proinflammatory postoperative status (also known as “acute postoperative frailty”) [83].

However, short-term T administration seems in turn to reduce systemic inflammatory cytokines including TNF-alpha, IL-6, and IL-1*β* [84] and to increase the anti-inflammatory cytokine IL-10 [85]. In a similar manner, magnesium seems to be fundamental in maintaining the threshold of antioxidant capacity and the control of oxidative stress [1]. Moreover, there is evidence of impaired overall antioxidant capacity and low-grade inflammation in magnesium-deficient cultured human and animals cells [86]. Inadequate intracellular magnesium may reduce the mitochondrial efficiency and increase the production of reactive oxygen species (ROS), determining structural and functional impairment of proteins [87] and DNA [88]. Interestingly, magnesium and T were found to be lower during systemic inflammation, and conditions associated with both increased ROS, oxygen-derived free radicals, oxygen peroxide production, and impaired antioxidant enzyme expression and activity [89, 90].

**Figure 2:**
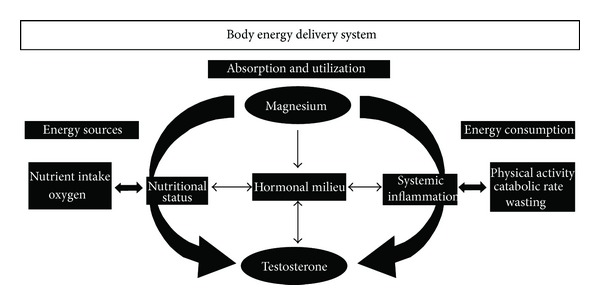
Relationship between Testosterone and magnesium, potential underlying mechanisms and clinical implications. The aging process is frequently characterized by an impaired homeostatic reserve due to an imbalance between energy assumption (left side) and utilization and consumption (middle and right sides). The activity of anabolic hormones, where T plays a central role, is influenced by mineral status (magnesium), along with caloric and protein intake. The systemic inflammation, which negatively influences magnesium and T and is in turn downregulated by these 2 factors.

Multiple changes in physiological pathways could be identified as pathogenic factors in several age-related phenomena including sarcopenia, frailty, disability, or altered immune response and chronic diseases. Therefore, during the aging process, where lower anabolic hormones, increased proinflammatory cytokines, and impaired nutritional status frequently coexist, combined strategies could have important clinical implications [91] (Figure 2).

## 8. Biomolecular Mechanisms Underlying the Relationship between Magnesium and Testosterone

In the recent years biomolecular interactions between T, SHBG, and magnesium have been studied by high performance liquid chromatography (HPLC) [92]. Excoffon and colleagues [92] provided evidence of a magnesium-mediated variation in the T-SHBG affinity. The change in magnesium levels inside the biological serum concentration range (0.75–0.95 mM) could lead to an enhancement of the Bio-T. In fact, the affinity of T to SHBG seems to change slightly with the magnesium concentration. Magnesium binds SHBG in a nonspecific mode, leading to an uncompetitive inhibition with T in binding SHBG and to a subsequent enhancement of Bio-T availability. The binding is accompanied by a magnesium release (or uptake) with a corresponding heat effect around in magnitude 17 kJ/mol [92].

SHBG is a homodimer comprising 373 amino acid residues for each monomer that transports the sex steroids in the blood and also regulating their activity in target cells [93]. Interestingly, each monomer of SHBG contains three metal-binding sites, one calcium-binding and two zinc-binding, [94, 95], that are divalent cations as well as magnesium. This data supports, at molecular level, the role of magnesium in modulating T bioactivity.

Guillaume's group investigated the role of magnesium on both the T-serum albumin binding process and the T displacement to its human serum albumin (HSA) binding cavity by DHEA. Serum albumin binds to T with low affinity [46]. In particular DHEA and T seem to bind to the same HSA site. DHEA has been shown to displace T to its HSA binding site. The authors observed *in vitro* that adequate magnesium concentrations displaced T from its HSA binding site [96, 97] and hypothesized the opportunity of testing *in vivo* the effects of magnesium supplementation, during DHEA treatment, on the Bio-T rate.

## 9. Conclusions

The ageing process seems to be at least partly due to the defect of anabolic hormones, low-grade inflammation, reduced physical activity, and a poor quality of nutrition.The permissive role that several micronutrients, such as magnesium, might exert on the serum concentration and the biological activity of T could be of undoubted interest for future clinical approaches. Male individuals with impaired magnesium status and T deficiency (accurately assessed) could benefit from magnesium and/or T treatment targeting physical performance. Future randomized clinical trials adopting synergistic treatments could lead to improving the effectiveness of T treatment, in preventing mobility limitation and adverse outcomes in older men.
